# Genetics of rheumatoid arthritis

**DOI:** 10.1007/s00281-022-00912-0

**Published:** 2022-01-27

**Authors:** Leonid Padyukov

**Affiliations:** Department of Medicine Solna, Division of Rheumatology, Karolinska Institutet and Karolinska Hospital, Stockholm, Sweden

**Keywords:** Rheumatoid arthritis, Autoimmunity, Autoantibody, Inflammation, Genetic polymorphism, HLA

## Abstract

Rheumatoid arthritis (RA) is an inflammatory autoimmune disease involving symmetric joints and is generally characterized by persistent pain, tenderness, and destruction of joints. The vast majority of RA patients produce autoantibodies, and immune cell involvement in disease development is well recognized, as is the contribution of other types of cells in synovial tissue, like fibroblasts. It is known that there are major genetic associations with the HLA locus, while multiple non-HLA genetic variants display relatively low risk of RA. Both HLA and non-HLA associations suggest that the profiles of genetic associations for autoantibody-positive vs. autoantibody-negative RA are different. Several alleles of *HLA-DRB1* are associated with high risk for autoantibody-positive RA, with the strongest risk characterized by valine at position 11 of the protein sequence (HLA-DRB1*04 and *10 alleles). There is a strong protective effect for the risk of autoantibody-positive RA associated with HLA-DRB1*13 alleles. Although major genetic associations have been known for several years, understanding of the specific mechanisms in the development of increased risk of RA for these variations is work in progress. Current studies focus on the binding of immune receptors involved in recognition of putative peptides in activation of T cells, as well as investigation of cell signaling mechanisms. At least a part of RA risk could be explained by gene–gene and gene-environment interactions. There are currently more than 150 candidate loci with polymorphisms that associate with RA, mainly related to seropositive disease, and new discoveries are anticipated in the future from investigation of diverse human populations. This new research will help create a strong foundation for the continuing process of integrating genetic, epigenetic, transcriptomic, and proteomic data in studies of RA.

Rheumatoid arthritis (RA) is a relatively common inflammatory disease in different world populations [1]. It does not generate immediate risk of death, such as with CVD or aggressive cancer, but significantly affects the everyday life of the patients and, together with unpleasant symptoms, may cause severe destruction of joints, multiple long-term side effects, and decreased life expectancy [2]. The cause of RA remains unknown, with the consensus of researchers being that multiple genetic and environmental factors are involved [3]. Therefore, large epidemiological studies to identify these factors have been performed, with a major focus on genetics. Historically, the first genome-wide association studies (GWAS) of RA were performed in 2007, the beginning of the GWAS era, in three parallel and separate studies: WTCCC, EIRA, and NARAC [4, 5]. During the following years, additional studies in several populations contributed to further discoveries of candidate genes, with the largest recent meta-analyses performed for 29,880 RA patients and 73,758 controls [6], for 22,628 RA patients and 288,664 controls [7], and recently for 35,871 RA patients and 240,149 controls [8], with notable overlap between these three studies. These data revealed a significant difference between the genetic effects of HLA haplotypes and non-HLA risk alleles, something which is an important attribute not only for RA, but of most autoimmune diseases.

RA is a phenotypically heterogeneous syndrome with diagnostic criteria based on clinical evaluation. The presence of autoantibody is of high value for diagnostical scores and may bias inclusion of patients to the study away from mild autoantibody-negative cases towards autoantibody-positive cases [9]. In a similar way to other complex diseases, sub-phenotyping of RA based on clinical and serological features is of great importance for interpretation of genetic associations. On the other hand, many genetic factors related to RA are also common for different autoimmune diseases, which strengthens the overall understanding of autoimmune mechanisms of human diseases and creates possibilities for future fine tuning of the diagnostic criteria.

Recent genetic analyses led to the discovery of more than 150 loci with association to RA, with HLA associations remaining most strong [6–8]. The HLA alleles were also found to be involved in multiple interactions with environmental and other genetic risk factors in the development of an increased risk of RA [10, 11]. A growing bulk of data from studies in the epigenetics, transcriptomics, and proteomics of RA requires proper integration with clinical data. It is also evident that the study of pre-disease is vital for understanding the early contribution of genetic risk factors in RA development, while studies of full-scale disease and more chronic cases may serve better for elucidating the involvement of genetic factors in treatment response and development of side effects and comorbidities.

One of the greatest challenges of genetic epidemiology studies is giving a functional interpretation from purely statistical evidence. Attribution of genetic associations to specific cellular or molecular phenotypes is not a straightforward process. Most previous genetic studies annotated associated SNPs by physical overlap with a particular gene or with a neighboring gene. With the development of our knowledge about human genome architecture, this type of annotation of signals has often been proven inaccurate and sometimes even misleading [12, 13]. Annotation requires broad assessment of the function of genes that are sometimes relatively distant from the association hit. The reasons for this confusion include linkage disequilibrium within genetic loci, but also the chromosome structure and complexity of chromatin function. With the advent of single-cell omics, a significantly more specific and robust annotation is expected in all areas of genetic research.

## Disease classification issues and autoantibodies in rheumatoid arthritis

As with any complex disease, RA represents a collection of symptoms, and the diagnosis is consensus based [9]. This may generate some uncertainty at initial stages of the disease and may cause misclassification due to overlap of symptoms between different rheumatic diseases. On the other hand, the study of only well-established chronic disease is likely to be biased towards individuals with more severe RA and to non-responders to treatment. The availability of specific biomarkers for early and accurate diagnosis significantly improves the quality of phenotyping for genetic research.

Rheumatoid arthritis is a prototype autoimmune disease that, together with other multiple impairments of the immune system, is characterized by development of different types of autoantibodies. Rheumatoid factor (RF) and anti-citrullinated peptide/protein antibodies (ACPA, usually measured by anti-CCP ELISA) are most commonly detected [2] and due to high predictive scores are a part of the classification criteria for RA [9]. Other types of autoantibodies to modified proteins are detected mostly in high correlation to RF and ACPAs [14–16]. In systemic screening of human population biobanks for these autoantibodies in different populations, it was found that they may be detected in blood long before the first symptoms of RA [17, 18]. Later, with development of symptoms, the concentration of ACPAs increases significantly and in the majority of future RA patients, ACPAs are present at least a few months before the onset of symptoms [19]. In an attempt to dissect the importance of different ACPA reactivities, several studies have been performed on a peptide array with broad selection of citrullinated and non-citrullinated peptides [20]. There is a great degree of cross-reactivity between different types of autoantibodies, although some of them cross-correlate less strongly. It became an accepted practice in rheumatology research to consider autoantibody status as an important covariate in predictive models. However, it is also true that there is only a moderate difference between ACPA-positive and ACPA-negative RA in important clinical features such as disease activity scores at baseline, age of onset, and sex distribution. The greatest difference is probably in the risk of bone erosion [15]. Regarding ACPA-positive RA, it is also characteristic that clinical phenotypes are very similar in subgroups of patients with significantly different concentrations of autoantibodies [21]. There have also been several attempts to correlate the presence of different autoantibodies to clinical phenotypes. Discrimination between ACP-positive and ACPA-negative RA was especially efficient in genetic studies. It has become evident in recent years that there is only a moderate overlap between genetic risk factors between these two subgroups of RA.

Misclassification due to similar clinical phenotypes is not uncommon in rheumatology research and may cause patients with other rheumatic diseases to be included in a study group. This is more common for study designs that lack accurate information about autoantibody status and, in general, is more common for ACPA-negative RA when this autoantibody status information is available. In these cases, the diagnosis may sometimes convert, with time, to spondyloarthritis, which is known to have a different genetic association pattern [22]. On the other hand, multiple pleiotropic effects have been detected between rheumatic diseases [23, 24].

Discoveries of genetic associations in RA arose in the past through investigation of large cohorts with relatively poor sub-phenotyping of RA. Better clinical characterization of RA subgroups should now be pursued.

## HLA-related genetic associations in rheumatoid arthritis

A genetic association between RA and variations at HLA was discovered early. With the growing understanding of the architecture of the HLA locus, in particular *HLA-DRB1*, it became evident that this association relates to a group of so named shared epitope (SE) alleles and is strongest for ACPA-positive RA [25]. The SE hypothesis was introduced in an attempt to find common features between different alleles or haplotypes within *HLA-DRB1*. Initially, a homologous amino acid sequence at positions 69–74 of the beta chain coded by the *HLA-DRB1* gene was identified as the protein structure responsible for association with RA. By following this hypothesis, several DRB1 haplotypes were combined in a single SE allele group in multiple studies and compared with a reference group of other haplotypes. HLA haplotypes from most of the alleles of DRB1*01, *04, and *10 groups represent SE alleles, with *14:02 later suggested as an important contributor for American populations. In contrast, HLA-DRB1*13 alleles were found to bring strong protection against RA [26]. With further sub-phenotyping of RA using autoantibodies, first RF and later with ACPA, it was found that the strongest associations are mainly with autoantibody-positive RA [27, 28].

### ACPA-positive RA

Recent revision of the shared epitope hypothesis revealed independent association hits in the HLA locus within and outside of *HLA-DRB1* [29]. The most profound risk effect (OR = 3.8 with HLA-DRB1*15 as a reference) was detected for individuals with amino acid valine (Val) at position 11 of the *HLA-DRB1* gene (or for highly linked histidine at position 13). The protein structure with Val at this position is characteristic both to HLA-DRB1*04 and HLA-DRB1*10 groups of alleles. Independent associations with lysine (Lys) at position 71 and alanine (Ala) at position 74 were also detected but could not be clearly explained by previously known associations of SE alleles since these amino acids are also common for several non-SE-related *HLA-DRB1* haplotypes. Glutamic acid (Glu) at position 71 seems to be most protective (corresponds to *13:01, *13:02, *13:04, and *01:03 alleles), although in cumulative amino acid association, it does not give a strong effect; this is likely to be due to admixture by *04:02 allele, which also carries Glu at this position but does not demonstrate protection from RA in this study. Additional associations were also detected with HLA-B, HLA-DPB1, and HLA-A for ACPA-positive RA, with significantly lower effect size: 2.12 (95%CI 1.89–2.38) for aspartic acid (Asp) at position 9 of HLA-B; 1.40 (95%CI 1.31–1.50) for phenylalanine (Phe) at position 9 of HLA-DPB1; and 0.85 (95%CI 0.81–0.90) for asparagine (Asn) at position 77 of HLA-A. These associations are mirrored by a highest peak in Manhattan plots for GWAS in multiple studies, including the first published WTCCC study where this hit was a single genome-wide significant one for RA [4]. The most significant difference in association of ACPA-positive RA between Asian and European populations is in the type of amino acid at position 11 of *HLA-DRB1* which generates the highest risk: in the Asian population, it is Asp, instead of Val, and this corresponds to the HLA-DRB1*09 group haplotype [30]. Additionally, in the Japanese population, a significant risk from allele of *HLA-DO* was found that was not seen in Europeans [30]. It is important to acknowledge that due to very high linkage disequilibrium within HLA, and specifically within *HLA-DRB1*, there are hundreds of correlated SNPs associated with RA. These associations are better explained by considering classical *HLA-DRB1* haplotypes and/or subsequent amino acid polymorphisms as described above. Until now, classical HLA haplotypes and amino acid sequences corresponding to these haplotypes have also played important roles in the functional interpretation of HLA associations with autoimmunity, including RA.

A recent study by an international RA genetic consortium (RACI) focused on the association of 19 types of ACPAs in RA patients with variations in the HLA locus [31]. Occurrences of different ACPA reactivities were highly correlated and it was suggested that the detected ACPAs should be split into two groups, canonical and non-canonical, based on reactivity in the bulk anti-CCP (citrullinated circular peptide) test. This stratification of RA individuals by type of ACPAs revealed different genetic architectures for the two groups: association of non-canonical autoantibodies with Asp at position 9 in HLA-B; expression of canonical antibodies mainly associated with Val at position 11 in *HLA-DRB1*. However, this stratification was far from being completely contrast and almost every RA patient with canonical autoantibodies was also positive to at least one of the non-canonical ones. It is also indicative that non-canonical autoantibodies directed to peptides from the same proteins as canonical, although to the different protein epitope. Later, in a separate study, it was shown that a similar association with Asp at position 9 in *HLA-B* is characteristic for the anti-carbamylated protein antibodies (anti-CarP) in RA patients in the absence of anti-CCP antibodies [32].

### ACPA-negative RA

Although association of ACPA-negative RA with the HLA locus is less pronounced, it was also recently studied in detail [33]. In this study, the authors performed additional filtering of patient clinical information to avoid misclassification through inclusion of patients with spondyloarthritis. It was shown that the profile of association of amino acids from *HLA-DRB1* locus is very different from its association with ACPA-positive RA. Highest risk was found for amino acids leucine (Leu) and serine (Ser) at position 11 of *HLA-DRB1*, with odds 1.22 compared to healthy controls. Interestingly, most of the subsequent classical haplotypes that carry these amino acids at position 11 of *HLA-DRB1* are either in linkage with *HLA-DRB3* or do not link to other *HLA-DRB* genes (HLA-DRB1*01). A later study of independent European cohorts suggested association of ACPA-negative RA with Leu at position 67 of *HLA-DRB1*, with a risk effect of 1.38 [34]; this result is likely due to contamination of the RA group with unrecognized ACPA-positive individuals since this amino acid is characteristic for all SE allele-positive haplotypes, including *14:02, and should be regarded with caution. Data from a Japanese population supports an association of ACPA-negative RA with position 71 at *HLA-DRB1*, which may subsequently reflect a protective effect of all haplotypes that do not carry arginine (Arg) at this position [30].

An independent association with similar effect size to *HLA-DRB1* was detected with asparagine (Asn) at position 9 of *HLA-B* in two independent studies; the association referred to classical allele HLA-B*08 [33, 34]. Association with HLA-DRB1*03 was previously found in minor studies for ACPA-negative RA, which also suggests a link to this haplotype with the *HLA-DRB3* gene or more generally with ancestral haplotype (AH) 8.1, with HLA-B*08 and HLA-DRB1*03 involved [35]. Both studies referred to here (Han et al. and Bossini-Castillo et al.) employed imputation of genetic variations at the HLA locus based on reference data collected by the Type 1 Diabetes Genetics Consortium, where *HLA-DRB* paralog genes are not well represented. Therefore, multiple associations found in this study, together with previous findings in the HLA locus, may suggest that at least some of these hits are in linkage disequilibrium with unappreciated polymorphisms within the genetic loci contributing to the extended AH 8.1 haplotype, including copy number variations in C4A/C4B locus and variations in *HLA-DRB3* gene.

### Non-European populations

The findings described above reflect HLA association mainly with RA in European populations, though the situation for Asian and African populations is not completely identical. For the majority of Asian populations, the association of seropositive RA with SE alleles is more specifically related to the HLA-DRB1*04:05 allele, which is not totally absent but is relatively rare in African and European populations. On the other hand, there are additional contributions from this locus which need further investigation in large patient groups. In independent studies of Japanese (ACPA defined) [30] and Korean (mixed by ACPA status) [36] RA patients, HLA association generally followed a pattern of polymorphisms very similar to that in Europeans. Extension of the spectrum of amino acid variations characteristic to HLA-DRB paralogs did not bring additional association signals for the Korean population. However, Japanese-specific association with HLA-DRB1*09 haplotype was repeatedly shown [30, 37]. In a study of Han Chinese ACPA-positive RA patients, strong association with Asp at position 160 of the *HLA-DQA1* gene was detected independent from the HLA-DRB1*04:05 allele and several other alleles from the HLA locus [38]. In an analysis of a multiethnic Malaysian cohort, a strong risk of ACPA-positive RA was associated with Val at position 11 of *HLA-DRB1*; it was found that HLA-DQB1*03:02 allele was inversely related to the risk of developing ACPA-positive RA in the Malay population [39].

Genetic association with RA in African populations remains significantly less investigated and awaits further research and evaluation. In relatively small studies with mixed serological phenotypes, a strong susceptibility in individuals with HLA-DRB1*04 alleles, or correlated SNPs, was confirmed, while other SE alleles were found less frequently in these population groups [40–42].

It is evident that genetic associations of HLA variants with different subgroups of RA and in different populations are the most important contributors to genetic risk of the disease. Further analysis of non-HLA genetic associations in the development of RA should be explicitly taken in the context of an understanding of this contribution.

## Non-HLA-related genetic associations in rheumatoid arthritis

**Fig. 1:**
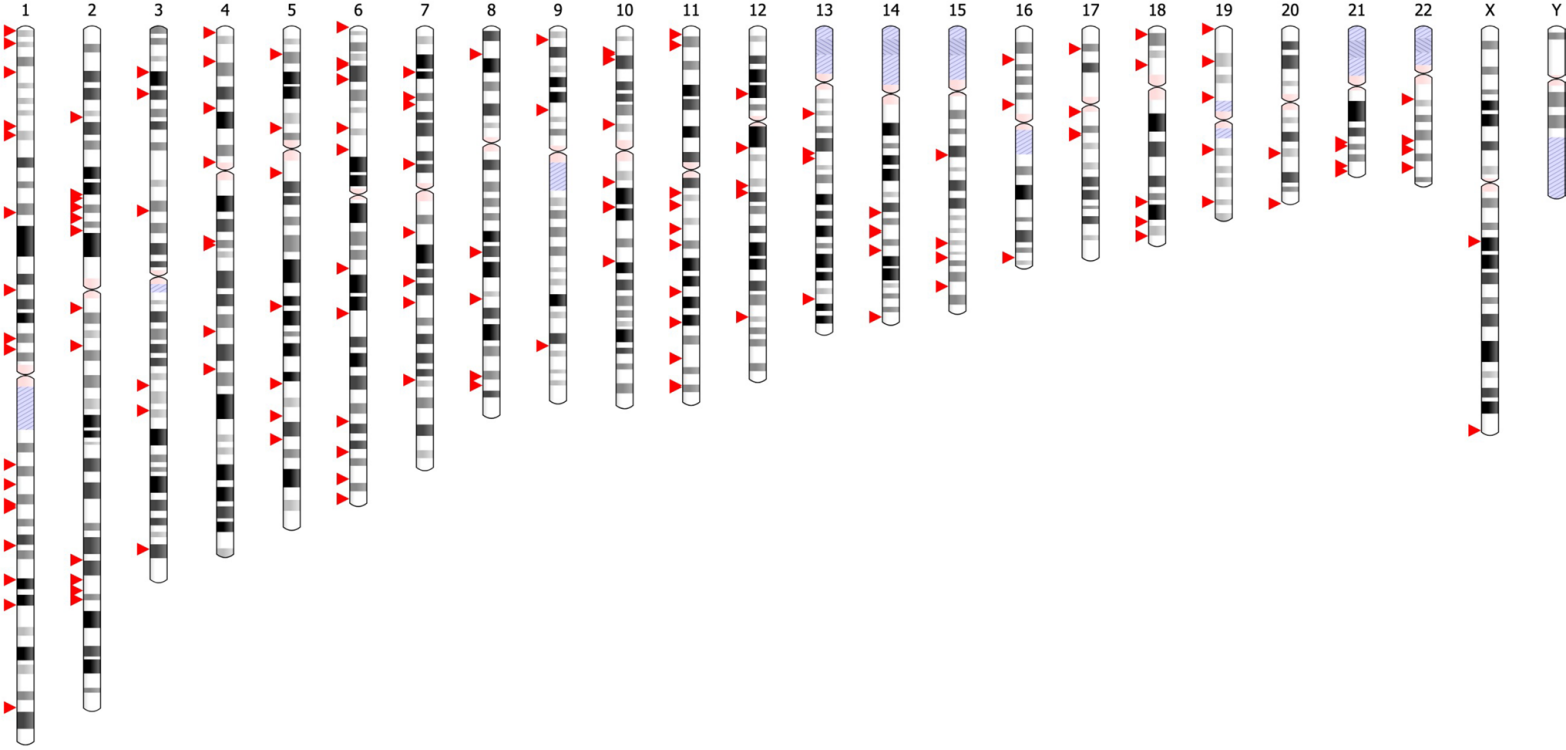
Physical position of SNPs in association with rheumatoid arthritis in human chromosomes

Several associations outside the HLA locus were detected before the GWAS era, including genetic variants at *PTPN22*, *CTLA4*, and *PADI4* genes [43–45]. With expanding data from GWAS from different populations, it became evident that the effect size of non-HLA associations is far lower in comparison to the major associations with *HLA-DRB1* SE alleles. Development of affordable genotyping methods, together with the use of transethnic meta-analysis, expanded this list to 151 loci and coverage of all human chromosomes except chromosome Y (Table 1, Fig. 1) [5–8, 34, 43, 46–56]. Not surprisingly, most associated hits (64% from the current list) could be annotated to genes with a known function in the immune system. The annotation to immune system is based on previous knowledge about specific function of the gene or a pathway in cells related to innate or adaptive immunity, or data on expression of this gene in immune cells. Many of these genes belong to immune system-specific or more ubiquitous cell signaling pathways. The involvement of these genes in cell function for non-immune cells, therefore, potentially extends the importance of genetic associations with RA to the function of cell types, tissues, and organs outside the immune system. One should keep in mind, however, that these annotations are usually based on the physical position of SNP within the gene location and that how gene function is linked to associated SNPs is a matter for future intensive research. Possible approaches will be discussed further in other articles in this issue.

**Table 1:** Non-HLA genetic associations with rheumatoid arthritis by December 2021

SNP^1^	Chromosome: position^2^	Gene/Locus	OR	95%CI	Immune function	ACPA	Population^3^	Reference
rs2843401, rs3890745, rs2258734	1:2596694	*MMEL1*	0.91	(0.89–0.93)	-	Positive	Asians, Europeans	[46, 50, 51]
rs227163	1:7901146	*TNFRSF9*	1.04	(1.02–1.06)	Yes	Combined	Asians	[6]
rs2240336, rs2301888, esv3585367	1:17347907	*PADI4*	0.88	(0.86–0.90)	Yes	Combined	Asians, Europeans	[8, 46, 50]
rs28411352	1:37812907	*MTF1*	1.10	(1.07–1.13)	-	Combined	Europeans, Trans Meta	[6]
rs883220, rs7540342	1:38151199	*POU3F1*	0.89	(0.86–0.92)	-	Positive	Europeans	[8, 46]
rs41269479	1:41701111	*HIVEP3*	1.15	(1.09–1.20)	Yes	Positive	Asians	[8]
rs4655698	1:67332644	*IL12RB2*	1.09	(1.05–1.11)	Yes	Positive	Trans Meta	[8]
rs41313373	1:92474854	*GFI1*	1.12	(1.08–1.16)	Yes	Positive	Europeans, Trans Meta	[8]
rs2476601	1:113834946	*PTPN22*	1.81	(1.73–1.89)	Yes	Positive	Europeans	[43, 52]
rs11586238, rs798000, rs624988	1:116720516	*CD2*	1.12	(1.09–1.16)	Yes	Positive	Europeans	[8, 51]
rs2228145, rs12126142	1:154454494	*IL6R*	0.93	(0.91–0.95)	Yes	Combined	Europeans, Trans Meta	[6, 7, 46]
rs2317230, rs2317231	1:157705207	*FCRL3*	1.08	(1.05–1.10)	Yes	Positive	Trans Meta	[6]
rs12026490	1:160447367	*SLAMF6*	0.80	(0.75–0.85)	Yes	Combined	Asians	[7]
rs3753389	1:160837363	*CD244*	1.30	(1.18–1.43)	Yes	Combined	Asians	[53]
rs10917571	1:161549621	*FCGR3A*	0.91	(0.89–0.94)	Yes	Positive	Trans Meta	[8]
rs2105325, rs61828284, rs6681482	1:173380586	*TNFSF4*	1.12	(1.08–1.15)	Yes	Combined	Europeans, Trans Meta	[6, 8, 57]
rs10911902, rs12145329	1:186663185	*PTGS2*	0.92	(0.89–0.95)	Yes	Positive	Trans Meta	[7, 8]
rs28398409	1:198614892	*PTPRC*	0.91	(0.88–0.94)	Yes	Combined	Trans Meta	[8]
rs762574969	1:235637057	*GNG4*	0.91	(0.88–0.94)	-	Combined	Europeans	[8]
rs10175798	2:30226728	*LBH*	0.92	(0.90–0.94)	Yes	Combined	Europeans, Trans Meta	[6]
rs34695944, rs13031237	2:60897715	*REL*	1.12	(1.08–1.15)	Yes	Positive	Europeans	[6, 47]
rs11900673	2:62225526	*B3GNT2*	1.15	(1.08–1.21)	Yes	Combined	Asians	[50]
rs6546146, rs934734, rs1858037	2:65329190	*SPRED2*	0.90	(0.88–0.93)	Yes	Positive	Asians, Europeans	[50, 52]
rs143259280	2:69982037	*PCBP1-AS1*	1.09	(1.06–1.12)	-	Positive	Trans Meta	[8]
rs6705628	2:73981235	*DGUOK-AS1*	0.88	(0.85–0.92)	-	Combined	Asians	[7]
rs10209110, rs12712065	2:100056230	*AFF3*	1.10	(1.08–1.13)	Yes	Combined	Asians, Europeans	[8, 46, 52]
rs6732565	2:110850255	*ACOXL*	1.07	(1.05–1.10)	-	Combined	Europeans, Trans Meta	[6, 8]
rs13426947, rs7574865	2:191068528	*STAT4*	1.16	(1.13–1.19)	Yes	Combined	Asians, Europeans	[46, 50]
rs10497813	2:198049348	*PLCL1-LINC01923*	1.06	(1.04–1.09)	-	Combined	Europeans, Trans Meta	[8, 58]
rs6715284, rs2141331	2:201289674	*CASP8*	1.15	(1.10–1.20)	Yes	Combined	Europeans, Trans Meta	[6, 8]
rs1980422	2:203745673	*CD28*	1.12	(1.09–1.16)	Yes	Combined	Europeans	[51]
rs11571302, rs3087243	2:203878211	*CTLA4*	0.88	(0.86–0.90)	Yes	Positive	Asians, Europeans	[8, 52]
rs77574423	3:11943270	*TAMM41-SYN2*	0.89	(0.86–0.93)	-	Positive	Europeans	[8]
rs4452313, rs4602367	3:17005540	*PLCL2*	0.93	(0.91–0.95)	Yes	Combined	Europeans, Trans Meta	[6, 7]
rs3806624	3:27723132	*EOMES*	0.92	(0.89–0.94)	Yes	Combined	Europeans, Trans Meta	[6, 7]
rs73081554	3:58317208	*DNASE1L3*	1.18	(1.11–1.25)	Yes	Combined	Europeans	[6]
rs62264113	3:127573490	*TPRA1*	0.92	(0.89–0.95)	-	Combined	Trans Meta	[8]
rs9826828	3:136683218	*IL20RB*	1.44	(1.28–1.61)	Yes	Combined	Europeans	[6]
rs4687070	3:189588861	*TPRG1-TP63*	1.15	(1.09–1.20)	-	Combined	Trans Meta	[8]
rs4690029	4:2721088	*FAM193A*	0.94	(0.92–0.96)	-	Combined	Trans Meta	[8]
rs13142500, rs13103285	4:10725733	*CLNK*	1.10	(1.08–1.13)	Yes	Combined	Asians, Trans Meta	[6, 7]
rs932036, rs874040, rs11933540	4:26089240	*RBPJ*	1.15	(1.11–1.19)	Yes	Combined	Europeans	[6, 46, 52]
rs2664035	4:48218822	*TEC*	1.07	(1.04–1.10)	Yes	Combined	Europeans	[6]
rs2867461	4:78592061	*ANXA3*	1.13	(1.09–1.17)	Yes	Combined	Asians	[50]
rs950918814	4:80031255	*ANTXR2*	0.93	(0.91–0.95)	-	Combined	Trans Meta	[8]
rs58107865	4:108140462	*LEF1*	0.84	(0.80–0.88)	Yes	Combined	Asians	[8]
rs6814280	4:122122507	*KIAA1109*	0.93	(0.90–0.96)	-	Positive	Europeans	[8]
rs2918392	5:10704685	*DAP*	0.94	(0.91–0.96)	Yes	Combined	Trans Meta, Europeans	[7, 8]
rs56787183	5:40499188	*PTGER4*	0.85	(0.80–0.90)	Yes	Combined	Trans Meta	[8]
rs71624119, rs6859212, rs7731626	5:56144903	*ANKRD55*	0.82	(0.79–0.85)	Yes	Combined	Asians, Europeans	[7, 8, 46, 52]
rs71624119, rs7731626	5:56144903	*ANKRD55*	0.85	(0.80–0.91)	Yes	Negative	Europeans	[8, 46]
rs2561477, rs403214, rs187579	5:103273223	*MACIR*	1.10	(1.06–1.13)	Yes	Combined	Europeans, Trans Meta	[6–8, 52]
rs657075, rs244685	5:132094425	*CSF2*	1.09	(1.06–1.12)	Yes	Combined	Asians, Trans Meta	[7, 50]
rs244468	5:143224856	*ARHGAP26*	0.93	(0.91–0.95)	-	Combined	Trans Meta	[8]
rs1422673	5:151059427	*TNIP1*	1.10	(1.06–1.14)	Yes	Positive	Europeans	[8]
rs9378815, rs6930468	6:426155	*IRF4*	0.91	(0.89–0.94)	Yes	Combined	Trans Meta	[6, 8]
rs12529514, rs12530098	6:14096427	*CD83*	1.15	(1.10–1.20)	Yes	Combined	Asians, Trans Meta	[7, 50]
rs113532504	6:15195451	*JARID2*	1.13	(1.08–1.18)	Yes	Combined	Europeans	[8]
rs67318457	6:23924793	*NRSN1*	1.09	(1.05–1.12)	-	Positive	Europeans	[8]
rs2234067, rs11420145	6:36387877	*ETV7*	1.15	(1.10–1.20)	Yes	Combined	Europeans, Trans Meta	[6, 8]
rs2233424, rs28362855	6:44266184	*POLR1C, NFKBE*	1.22	(1.17–1.26)	Yes	Combined	Asians, Europeans	[6, 8, 50]
rs72928038	6:90267049	*BACH2*	1.09	(1.06–1.11)	Yes	Combined	Europeans, Trans Meta	[8, 23, 59]
rs548234, rs9372120, rs3804333	6:106120159	*PRDM1-ATG5*	1.11	(1.07–1.15)	Yes	Positive	Asians, Europeans	[6, 8, 51]
rs10499194, rs6920220, rs6932056, rs7752903	6:137681500	*TNFAIP3*	1.33	(1.26–1.40)	Yes	Positive	Asians, Europeans	[6, 46, 50, 60]
rs9373594	6:149513438	*PPIL4*	1.09	(1.06–1.12)	Yes	Combined	Asians	[6]
rs629326, rs2451258	6:159075681	*TAGAP*	1.11	(1.08–1.14)	Yes	Positive	Europeans	[6, 46]
rs59466457, rs3093023, rs1571878	6:167124266	*CCR6*	0.86	(0.84–0.88)	Yes	Positive	Asians, Europeans	[46, 50, 52]
rs940825	7:17167540	*AGR3-AHR*	1.13	(1.08–1.18)	-	Positive	Europeans	[8]
rs182199544	7:27044962	*SKAP2-HOXA1*	0.87	(0.84–0.91)	Yes	Positive	Europeans	[8]
rs67250450, rs740122	7:28135367	*JAZF1*	0.92	(0.90–0.95)	-	Combined	Europeans, Trans Meta	[6, 7]
rs6583441	7:50322278	*IKZF1*	0.95	(0.93–0.97)	Yes	Combined	Trans Meta	[8]
rs113066392, rs73366469	7:74611832	*GTF2IRD1-NCF1*	1.43	(1.33–1.55)	Yes	Combined	Asians	[7, 56, 61]
rs4272, rs42044	7:92607515	*CDK6*	0.92	(0.89–0.94)	Yes	Combined	Europeans, Trans Meta	[6, 8]
rs6979218	7:100295525	*CASTOR3-SPDYE3*	0.92	(0.89–0.94)	-	Combined	Europeans, Trans Meta	[8]
rs3807306, rs10488631	7:128940626	*IRF5*	0.88	(0.86–0.91)	Yes	Combined	Asians, Europeans	[46, 50, 52]
rs4840565, rs2736340	8:11488036	*BLK*	1.12	(1.09–1.15)	Yes	Combined	Asians, Europeans	[6–8, 47]
rs998731, rs10453119	8:80183160	*TPD52*	1.08	(1.05–1.11)	-	Combined	Europeans, Trans Meta	[6, 8]
rs678347, rs1264600	8:101451374	*GRHL2*	1.08	(1.05–1.11)	-	Combined	Europeans, Trans Meta	[6, 8]
rs1516971, rs16903108	8:128529854	*PVT1*	1.15	(1.10–1.20)	-	Combined	Europeans, Trans Meta	[6, 8]
rs11777380	8:133199722	*CCN4*	0.92	(0.90–0.95)	-	Combined	Trans Meta	[8]
rs911760	9:5438435	*PLGRKT*	1.15	(1.09–1.20)	-	Positive	Europeans	[8]
rs2812378, rs11574914	9:34710263	*CCL21*	1.12	(1.09–1.16)	Yes	Positive	Europeans, Trans Meta	[6, 8, 46, 52]
rs3761847, rs10985070	9:120927961	*TRAF1-C5*	0.92	(0.90–0.95)	Yes	Positive	Europeans, Trans Meta	[5, 6]
rs706778, rs3134883	10:6056986	*IL2RA*	1.10	(1.08–1.13)	Yes	Combined	Europeans, Trans Meta	[6, 7, 52]
rs947474, rs502919	10:6348488	*PRKCQ*	0.92	(0.90–0.94)	Yes	Combined	Europeans, Trans Meta	[6, 7]
rs2275806, rs3824660, rs10905284	10:8053377	*GATA3*	0.93	(0.91–0.95)	Yes	Positive	Europeans, Trans Meta	[6, 8, 46]
rs793108, rs793095, rs1538981	10:31126177	*ZNF438*	1.07	(1.05–1.09)	-	Combined	Trans Meta	[6–8]
rs2671692, rs7097397	10:48889774	*WDFY4*	0.92	(0.90–0.94)	Yes	Combined	Asians, Trans Meta	[6–8]
rs12764378, rs10821944, rs7902146	10:62040245	*ARID5B*	1.16	(1.13–1.19)	Yes	Positive	Asians, Europeans	[8, 46, 50]
rs6479800, rs3125734	10:62277122	*RTKN2*	1.11	(1.07–1.15)	-	Combined	Asians, Trans Meta	[6, 49]
rs726288	10:79947217	*SFTPD*	1.14	(1.07–1.20)	Yes	Combined	Asians	[6]
rs734094	11:2301990	*TSPAN32*	1.08	(1.05–1.10)	Yes	Positive	Trans Meta	[8]
rs9943599	11:9731194	*SWAP70*	1.09	(1.06–1.11)	Yes	Combined	Asians, Trans Meta	[7, 48]
rs595158, rs968567, rs7943728	11:61142109	*FADS1-FADS2-FADS3*	1.12	(1.07–1.16)	-	Combined	Europeans	[6, 8, 46]
rs660442, rs479777	11:64275525	*BAD*	0.90	(0.87–0.93)	Yes	Combined	Asians, Trans Meta	[7, 8]
rs59578717	11:69092402	*TPCN2*	0.91	(0.88–0.94)	-	Combined	Asians	[7]
rs3781913, rs79145843	11:72662452	*PDE2A-ARAP1*	0.88	(0.84–0.91)	Yes	Combined	Asians, Trans Meta	[7, 8, 50]
rs4409785	11:95578258	*CEP57*	0.91	(0.88–0.94)	-	Combined	Europeans, Trans Meta	[6, 7]
rs138193887	11:108096623	*CUL5*	1.21	(1.13–1.29)	-	Combined	Europeans	[6]
rs10892279, rs4938573, rs73005423	11:118741072	*DDX6*	1.15	(1.09–1.22)	-	Positive	Asians, Europeans	[8, 46, 50]
rs10790268	11:118858682	*CXCR5*	0.87	(0.84–0.90)	Yes	Combined	Europeans	[6]
rs12795702	11:128286419	*LOC107984408*	1.09	(1.06–1.12)	-	Combined	Trans Meta	[7]
rs73013527, rs10556591	11:128627057	*FLI1-ETS1*	0.92	(0.90–0.95)	Yes	Combined	Trans Meta	[6, 8]
rs4963581	12:24660347	*LOC105369698*	1.09	(1.06–1.12)	-	Combined	Trans Meta	[7]
rs1427749	12:45976333	*SCAF11*	1.08	(1.05–1.11)	Yes	Combined	Trans Meta	[8]
rs773125, rs4622308, rs705700	12:56001170	*CDK2*	1.09	(1.07–1.12)	Yes	Combined	Europeans, Trans Meta	[6, 7]
rs10683701, rs1696466	12:57698305	*OS9-AGAP2*	1.06	(1.04–1.08)	-	Positive	Europeans, Trans Meta	[8, 46]
rs10774624, rs77465633, rs3184504	12:111395984	*SH2B3-PTPN11*	1.32	(1.20–1.45)	Yes	Combined	Asians, Europeans	[6, 7]
rs61944750	13:28060796	*FLT3*	0.91	(0.88–0.94)	Yes	Combined	Trans Meta	[8]
rs9603616, rs9532434	13:39793932	*COG6*	0.89	(0.87–0.91)	-	Combined	Asians, Europeans	[6, 7]
rs2147161	13:42408166	*AKAP11-LINC02341*	1.10	(1.06–1.13)	-	Positive	Europeans	[8]
rs9557321	13:99868847	*CLYBL*	1.73	(1.42–2.11)*	-	Negative	Europeans	[34]
rs3783782, rs146492555	14:61473957	*PRKCH*	1.14	(1.09–1.18)	-	Combined	Asians, Trans Meta	[6, 8]
rs1950897, rs1885013	14:68293424	*RAD51B*	1.11	(1.08–1.14)	-	Combined	Asians, Europeans	[6, 7]
rs3825568	14:68793871	*ZFP36L1*	1.08	(1.06–1.11)	-	Combined	Asians, Trans Meta	[7, 48]
rs175714	14:75515513	*BATF*	0.94	(0.92–0.96)	Yes	Combined	Trans Meta	[8]
rs2841277, rs2582532, rs3001423	14:104924668	*PLD4*	0.85	(0.82–0.88)	-	Combined	Asians	[6, 8, 50]
rs8043085, rs8032939, rs6495979	15:38535939	*RASGRP1*	0.88	(0.86–0.90)	Yes	Positive	Asians, Europeans	[6–8, 46]
rs8026898	15:69699078	*TLE3*	1.14	(1.11–1.17)	Yes	Combined	Europeans, Trans Meta	[8, 46]
rs115284761	15:77034495	*PSTPIP1*	0.91	(0.89–0.94)	Yes	Combined	Europeans, Trans Meta	[8]
rs199894206, rs7171617	15:90495653	*IQGAP1*	1.12	(1.08–1.16)	-	Combined	Asians, Trans Meta	[7, 8]
rs4780401, rs7206670, rs4584833	16:11745470	*TXNDC11*	1.07	(1.05–1.10)	-	Combined	Europeans, Trans Meta	[6, 7]
rs149041927	16:23859897	*PRKCB*	0.91	(0.88–0.94)	Yes	Combined	Asians	[7]
rs12918327, rs34480360	16:30615295	*ZNF689*	1.09	(1.06–1.12)	-	Combined	Trans Meta	[7, 8]
rs13330176, rs9927316	16:85985481	*IRF8*	0.91	(0.89–0.94)	Yes	Positive	Asians, Europeans	[6–8, 46]
rs72634030, rs8073171	17:5369285	*C1QBP*	1.12	(1.08–1.17)	Yes	Combined	Trans Meta	[6, 8]
rs11375064	17:27577049	*KSR1*	0.93	(0.90–0.95)	-	Positive	Trans Meta	[8]
rs1877030	17:39583908	*MED1*	1.09	(1.06–1.12)	-	Combined	Trans Meta	[6]
rs2305480, rs2872507, rs56750287	17:39905943	*GSDMB*	0.93	(0.91–0.95)	Yes	Combined	Europeans, Trans Meta	[6, 8, 46]
rs591549	18:3542249	*DLGAP1*	0.91	(0.88–0.94)	-	Combined	Trans Meta	[8]
rs2847297, rs8083786, rs7241016	18:12797695	*PTPN2*	0.91	(0.89–0.94)	Yes	Combined	Asians, Europeans	[6, 8, 50]
rs866205108	18:62342401	*TNFRSF11A*	1.10	(1.06–1.14)	Yes	Positive	Trans Meta	[8]
rs2469434, rs143107126	18:69876810	*CD226*	1.07	(1.05–1.10)	Yes	Combined	Asians, Trans Meta	[6, 8]
rs1943199	18:75750899	*LINC01898*	1.94	(1.54–2.44)*	-	Negative	Europeans	[34]
rs10415976	19:941603	*ARID3A*	0.92	(0.90–0.95)	-	Combined	Trans Meta	[8]
rs34536443	19:10352442	*TYK2*	0.68	(0.62–0.75)	Yes	Positive	Europeans	[46]
rs147622113	19:10661265	*ILF3*	0.68	(0.60–0.77)	Yes	Combined	Europeans	[6]
rs55762233	19:19256510	*HAPLN4*	1.10	(1.07–1.14)	-	Combined	Trans Meta	[8]
rs28373672	19:35722170	*KMT2B*	0.93	(0.91–0.96)	-	Combined	Trans Meta	[8]
rs8106598	19:51514686	*SIGLEC6*	1.08	(1.05–1.11)	-	Positive	Trans Meta	[8]
rs6032662, rs4810485, rs1883832	20:46105671	*CD40*	0.90	(0.88–0.92)	Yes	Positive	Asians, Europeans	[6, 8, 46, 51]
rs6011186, rs4809371	20:63852655	*C20orf181*	0.90	(0.87–0.93)	-	Combined	Asians, Trans Meta	[7, 8]
rs73194058, rs8126756, rs2300373	21:33391982	*IFNGR2*	1.09	(1.06–1.12)	Yes	Combined	Europeans, Trans Meta	[6–8]
rs9979383, rs8133843, rs66922517	21:35343463	*RUNX1*	1.08	(1.06–1.11)	Yes	Combined	Europeans, Trans Meta	[6–8, 46]
rs1893592	21:42434957	*UBASH3A*	1.10	(1.07–1.13)	Yes	Combined	Europeans, Trans Meta	[6]
rs2075876, rs7278257, rs11454989	21:44289270	*AIRE*	0.91	(0.89–0.94)	Yes	Combined	Asians, Trans Meta	[7, 8, 54]
rs11089637, rs5754104	22:21624807	*UBE2L3-YDJC*	1.09	(1.06–1.12)	Yes	Combined	Europeans, Trans Meta	[6–8]
rs5756407	22:36920217	*CSF2RB-LOC105373023*	1.06	(1.04–1.08)	Yes	Combined	Trans Meta	[8]
rs909685, rs2069235	22:39351666	*SYNGR1*	1.14	(1.11–1.17)	-	Combined	Asians, Europeans	[6, 7]
rs35156883	22:45350272	*SMC1B*	1.10	(1.06–1.13)	-	Combined	Asians	[7]
rs201408742, rs6619397, rs74842123	X:79209119	*GPR174-KIF4CP*	1.11	(1.08–1.15)	Yes	Combined	Asians, Trans Meta	[6–8]
rs13397, rs5987194	X:153982797	*IRAK1*	1.15	(1.12–1.18)	Yes	Positive	Asians, Europeans	[6, 7, 46]

^1^Genetic variation with genome-wide significance for association

^2^Position is indicated for the first variation in the list

^3^Trans Meta – meta-analysis for populations with European and Asian ancestry

*OR *odds ratio, *95%CI* 95% confidence interval for OR, *ACPA *anti-citrullinated peptide/protein antibodies

As is the case with the HLA locus, genetic association studies that focus on autoantibody status reveal remarkable differences between ACPA-positive and ACPA-negative arthritis. However, previous investigations were most often performed in RA patients without known autoantibody status and the differences remain non-confirmed by direct testing in the subgroups of RA. According to follow-up analysis in different cohorts all over the world, it is likely that ACPA-positive RA is more prevalent and therefore most of the association hits in studies without discrimination by serology represent associations with autoantibody-positive RA. There have been several attempts to perform GWAS separately for these two groups that often end up with replication of hits for ACPA-positive RA and only few association signals for ACPA-negative RA (Table 1).

The number of known genetic associations with RA is ever growing due to an increase in the number of observations and the resulting increase of statistical power. To expand this further, the complementary approach is in the investigation of population groups to capture differences due to ancestral, most often ethnic, background. In a similar way to the situation for HLA genetics until recently, studies of Europeans have been dominant, and it is important to extend investigation of the scope of human polymorphisms specific for different populations in association studies. These studies are in progress for major Asian and African populations and will help to map true genetic associations for RA.

From the available data in European and East Asian populations, there are several genetic associations that show population differences. It was found that the second-best association with RA in the *PTPN22* gene with exonic SNP rs2476601 is not detectable in East Asian populations due to very low frequency of the polymorphism in these populations [50]. Similarly, several other SNPs associated with RA in European populations close to the *IL20RB*, *CUL5*, *TYK2*, and *ILF3* loci are not polymorphic or have very low allelic frequency in East Asian populations [6, 7]. In contrast, association with RA for the SNPs rs12026490 close to *SLAMF6* was not reproduced in Europeans due to a < 1% frequency of the risk allele [7]. One may expect more population-specific association hits to be discovered in the future. Currently, 18% of known associations in European populations are not confirmed for Asian populations, while only 16% independently reach convincing genome-wide significance in both populations. On the other hand, comparing the effect size for the majority of associated SNPs from trans-ethnic meta-analyses reveals no differences, not only in the presence of significant associations in two different population, but also when one of the groups did not reach genome-wide significance for association [6, 7].

As mentioned previously, the data regarding non-HLA genetic associations with seronegative RA is less consistent and robust. This subgroup is usually a smaller fraction of large GWAS of RA and until recently, only three confirmed associations have demonstrated genome-wide significance (Table 1), close to loci *ANKRD55*, *IRF4*, and *LINC01898* [34, 46, 55]. At least some of these associations match to hits for association with ACPA-positive RA and, therefore, may also occur due to misclassification within the seronegative group, as discussed above. Most recent trans-ancestry meta-analysis did not confirm genome-wide significant association for seronegative RA [8]. Therefore, GWAS of ACPA-negative RA will be an emerging area of research, although it requires an increase in the size of studies and better phenotypic data for this RA subpopulation.

Common genetic risk factors for different autoimmune diseases were found previously and investigated specifically with Immunochip genotyping [62]. Subsequently, several studies confirmed pleiotropic effects from these genes and by cross-investigation of autoimmune diseases discovered possible new associations for RA [23, 24].

**Fig. 2:**
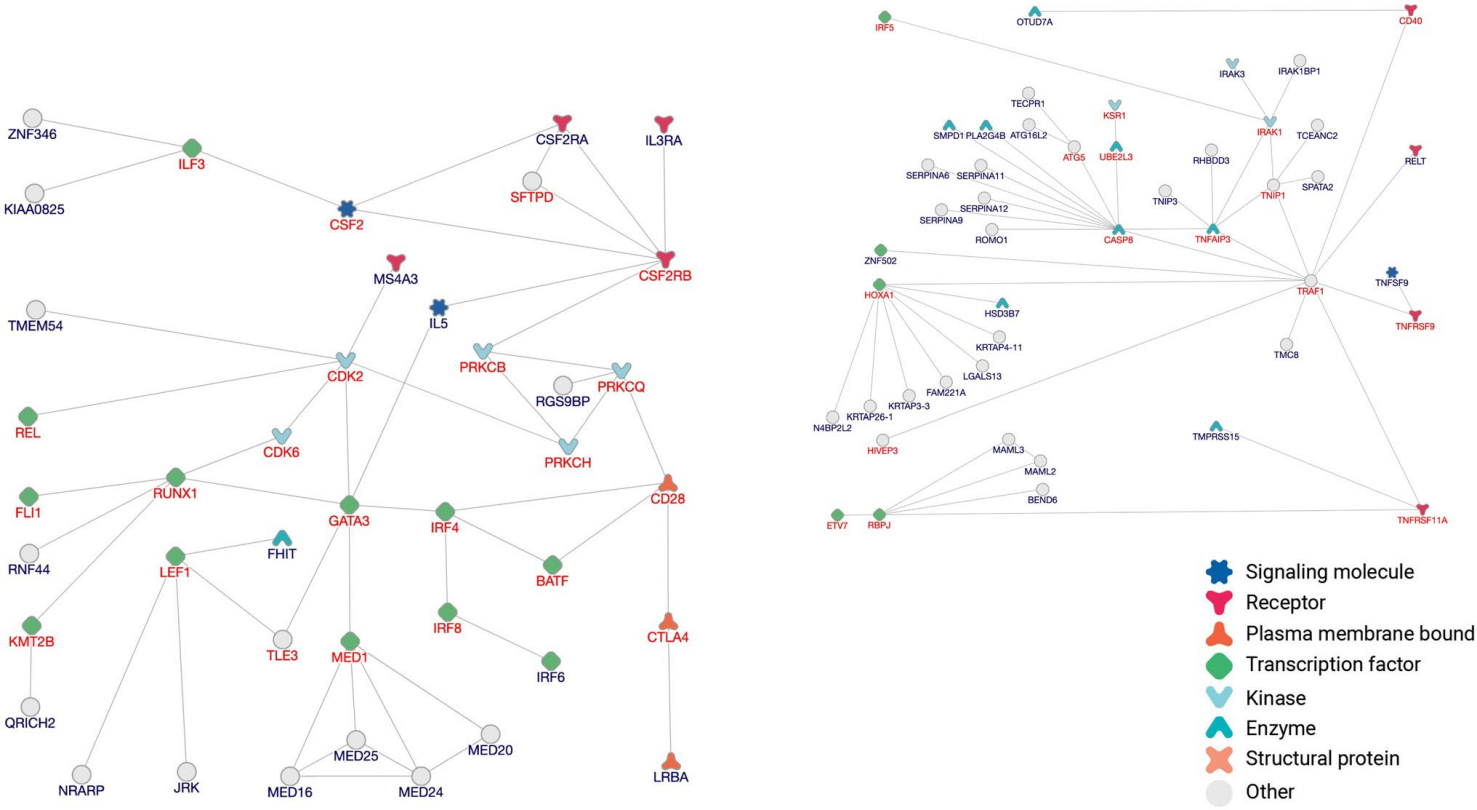
PPI networks for the genes associated with rheumatoid arthritis

**Fig. 3:**
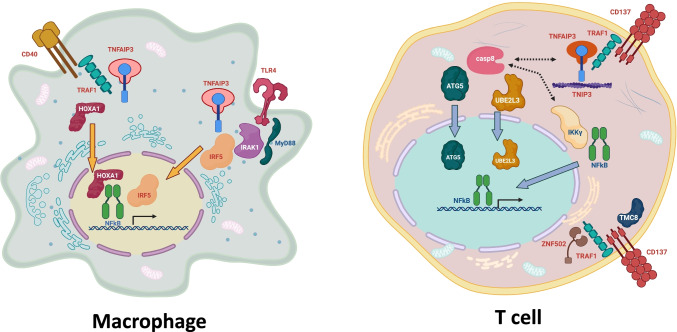
Hypothetical cell pathways based on PPI networks for some of the genes associated with rheumatoid arthritis

Further integration of genetic association data is required, together with available omics data. Our PPI analysis using inBio Discover (INTOMICS, Denmark) of the gene list from Table 1 generated at least two comprehensive networks with only direct PPI and demonstrated possible involvement in the generation of RA risk for several signaling pathways in immune cells (Figs. 2 and 3).

More analyses in serologically stratified RA cohorts and in populations with different ancestries are required to elucidate genetic risk of RA using data from genetic association studies.

## Gene–gene and gene-environment interaction in risk of rheumatoid arthritis

An investigation of genetic risk factors for common complex diseases should consider the phenomena of gene-environment and gene–gene interaction. There are several statistical approaches for detecting interactions and when discussing interaction, it is important to clarify which definition of interaction is being used. Most often, interaction is part of a statistical model that tests for multiplicativity of contribution for selected parameters in the development of disease risk. The null hypothesis is that the coefficient for interaction mode in the regression model is null. In terms of odds ratio to develop disease, this transfers to the expectation that in a group of individuals with a combination of two parameters, the odds ratio will represent the product of odds for separate parameters. Deviation from the null hypothesis will be the indication for “multiplicative interaction.” In epidemiological studies following the Rothman hypothesis [63], the null hypothesis based on expectation for additivity of odds ratios for a group of individuals with a combination of two parameters was compared to groups with only one of these risk factors. Therefore, “additive interaction” arises in the case of significant deviation from the sum of contribution of each risk factor.

In previous studies, considerable interaction between cigarette smoking and SE alleles was detected in generation of risk, first for RF positive [10], and later for ACPA-positive RA [64]. Further analyses of other environmental factors for interaction with these alleles were performed and it was confirmed for independent Asian, African American, and European populations [65–71]. Several other studies of environmental effects, including exposure to textile dust and alcohol consumption, also found interaction with SE alleles [72, 73]. Additionally, two genetic loci, genes *GSTT1* and *HMOX1*, at chromosome 22 were identified to increase risk of RA due to interaction with smoking. These genes are functionally related to protection from oxidative stress [74].

Several studies of gene–gene interaction in the development of risk of ACPA-positive RA were performed previously, with *PTPN22* risk allele and variation close to *MAP2K4* gene found to interact with SE alleles [75, 76]. In another study, gene–gene interaction between variations in the *BANK1* and *BLK* genes from chromosomes 4 and 8, respectively, was shown to increase risk of RA [77]. Based on our dominion hypothesis [72], we performed a study showing the global effect of RA risk alleles in interaction with SE alleles on the development of additional risk for ACPA-positive RA [11]. Although this study was initially criticized for statistical biases [78], careful statistical modeling has confirmed that it does indeed describe true effects and could be applied to study gene–gene interactions in other binary phenotypes with a known dominant risk factor [79]. Therefore, the conclusion of this study is that significant interaction between the *HLA-DRB1* SE alleles and the group of SNPs associated with ACPA-positive RA is a global feature that increases risk of ACPA-positive RA. It was also shown in this study that gradual decrease of the number of interacting risk alleles in a group of individuals with *HLA-DRB1* SE alleles will decrease the risk for RA. No gene–gene or gene-environment interactions in development of the risk of ACPA-negative arthritis have so far been found. Further analysis of statistical interactions may help to integrate functionally relevant associations involved in the development of RA.

## DNA methylation in association with rheumatoid arthritis

Development of tools for fast analysis of a massive number of methylation sites, like Illumina 450 and Illumina EPIC arrays, opened the possibility for genome-wide epigenetic study (GWES). To date, the largest GWES of RA was performed on whole blood genomic DNA of 354 ACPA-positive RA and 357 healthy controls [80]. Epigenetic changes at disease baseline are massive and obviously non-specific, reflecting basic inflammatory reactions. In an attempt to identify differentially methylated sites (DMS) that are more specifically related to RA development, genetic association signals for RA were integrated with data for DMS in genomic DNA from whole blood in ACPA-positive RA. This approach helped to focus only on a few DMS that are regulated by SNPs with association to the disease. Interestingly, almost all these DMS, 9 out of 10, were located within the HLA locus. Additionally, following known association of seropositive RA with cigarette smoking, one of these DMS, close to the HLA locus, was attributed as a major contributor to the risk of ACPA-positive RA due to gene-environment interaction [81]. However, DNA methylation profile reflects contribution of the multiple environmental factors and of the ongoing inflammation in RA patients and it is difficult to identify RA-specific casual relations between these factors and DMS changes. It was later shown that methylome of peripheral mononuclear cells can be used to anticipate the evolution of undifferentiated arthritis to RA, and these modifications could be annotated to a number of inflammatory pathways and transcription factors [82]. The significance of ACPAs during RA development was analyzed in serologically distinct RA subgroups in a study of twins discordant for ACPA status [83]. However, only marginal differences in the methylation profile of whole blood DNA of twins were found when data was corrected for cell composition; this highlights the importance of blood cell profiling in epigenetic studies of RA development. With the goal of determining RA-specific methylation events, several research groups conducted epigenetic studies with profiling of specific cell subpopulations. These studies demonstrated the following: significant changes in methylome of CD4 + memory cells compared to CD4 + naïve cells [84]; specific methylation-induced regulation of *CTLA4* promoter [85] and *FOXP3* enhancer in Tregs [86]; evidence for epigenetic regulation of inflammatory cytokines in monocytes [87]; and multiple immune-related pathways in fibroblast-like synoviocytes (latterly, in comparison with osteoarthritis) [88] in RA. Interestingly, a common pattern of epigenetic changes was found in a regulation of interferon-related genes for different autoimmune diseases, including RA, with hypomethylation of these genes in CD4 + cells being highly predictive to disease development [89].

While epigenetic studies most commonly focus on the available pool of peripheral blood cells, significant diversity was shown for the tissue from anatomically different joints. Synovial fibroblasts of different anatomical origins represent different transcriptomes that translate into unique joint-specific phenotypes of these cells, which is likely to influence methylation profile in RA with different localization [90].

Coming studies of methylome in RA should consider the important differences observed in genetic studies between the RA subgroups. It is desirable to build the experimental design either on stratification of seropositive and seronegative RA, or to adjust the differential methylation analysis for serological status. The major challenge for the analysis is in a study design in which healthy controls are not necessarily the optimal reference group. Epigenetic studies should be integrated with genetics, transcriptomics, and proteomics to reveal a true pattern of pathway modulation in the development of RA.

## Conclusion

Multiple genetic polymorphisms contribute to predisposition to RA, and this is best investigated in seropositive disease. More detailed studies of seronegative RA are pending. As for many autoimmune diseases, the effect size for risk of seropositive RA is dominated by several *HLA-DRB1* alleles. Although there are a few success stories with regard to translation of genetic studies of RA into the biological functions, most of the associations remain to be better interpreted and tested in the future. The major challenge will be the integration of genetic association studies with epigenetics, transcriptomics, and proteomics to produce an understanding that will enable personalized medical help and, ultimately, prevention of severely incapacitating chronic RA.
